# Supplementary material for: Divergent regulation of basement membrane trafficking by human macrophages and cancer cells
Source: Nat Commun. 2022 Oct 27;13:6409. doi: 10.1038/s41467-022-34087-x (PMC9613642; doi:10.1038/s41467-022-34087-x)
Supplement: Supplementary file 1 — Supplementary Information [file 41467_2022_34087_MOESM1_ESM.pdf]

Supplementary Information

Divergent Regulation of Basement Membrane Trafficking by Human Macrophages and Cancer Cells

Julian C. Bahr[1,2*], Xiao-Yan Li[2,3,4*], Tamar Y. Feinberg[2,3,4], Long Jiang[2,3,4], and Stephen J. Weiss[1,2,3,4]

[1]Cancer Biology Graduate Program, University of Michigan, Ann Arbor, MI 48109, USA.

[2]Life Sciences Institute, University of Michigan, Ann Arbor, MI 48109, USA.

[3]Division of Genetic Medicine, University of Michigan, Ann Arbor, MI 48109, USA.

[4]Department of Internal Medicine, University of Michigan, Ann Arbor, MI 48109, USA.

Supplementary Figure 1

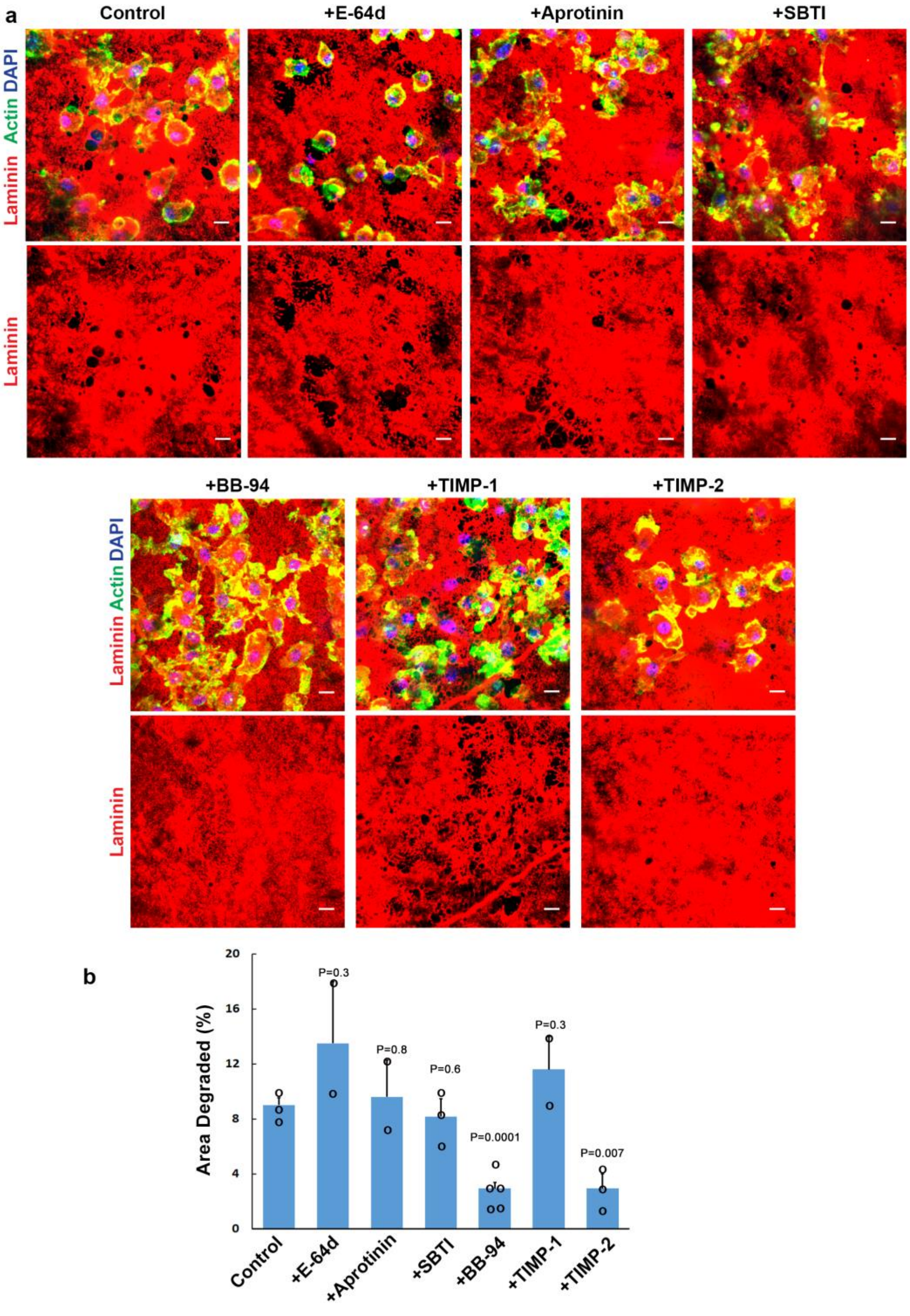

Supplementary Figure 1: Mouse macrophages require matrix metalloproteinases for basement membrane remodeling. (A) Macrophages were cultured atop basement membrane constructs for 6 d with LPS (1 µg/mL) in the absence or presence of inhibitors directed against cysteine proteinases (100 µM E-64d), serine proteinases (100 µg/mL aprotinin; 100 µg/mL soybean trypsin inhibitor, SBTI), matrix metalloproteinases (5 µM BB-94), 12.5 µg/mL TIMP-1 or 5 µg/mL TIMP-2. Results are representative of 3 independent experiments. (B) The percent basement membrane degradation is quantified with results expressed as the mean ± SEM (with n=3, 2, 2, 3, 5, 2 and 3 independent exps for each of the listed variables, respectively) with significance determined by two-tailed t test.

Supplementary Figure 2

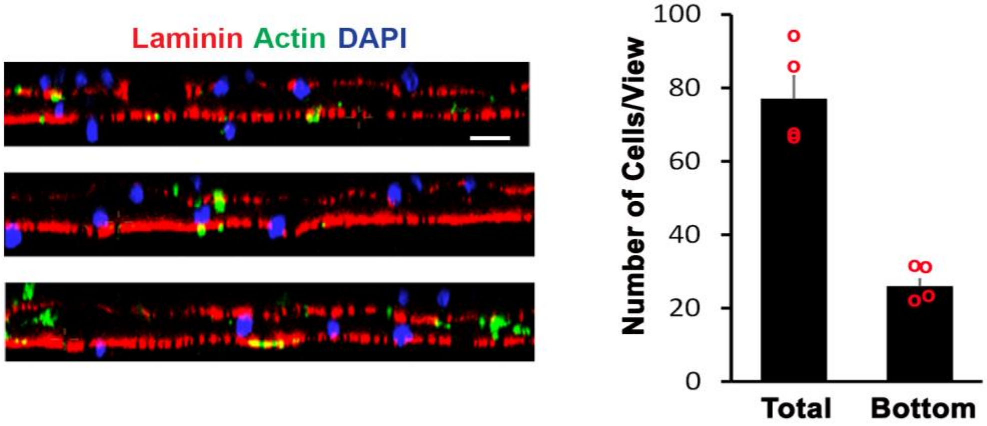

Supplementary Figure 2: Transmigrating macrophages penetrate both the apical and basal membrane surfaces. Orthogonal images (3 representative images shown) of actin- (green) and DAPI- (blue) labeled mouse macrophages atop laminin-labeled (red) explants. Approximately 30% of all macrophages counted in 3 or more independent fields actively penetrate the basal aspect of the basement membrane. Results are expressed as the mean ± SEM (n = 3 and 4 independent experiments for number of cells atop the upper basement membrane of below the basal basement membrane). Bar: 10 µm.

Supplementary Figure 3

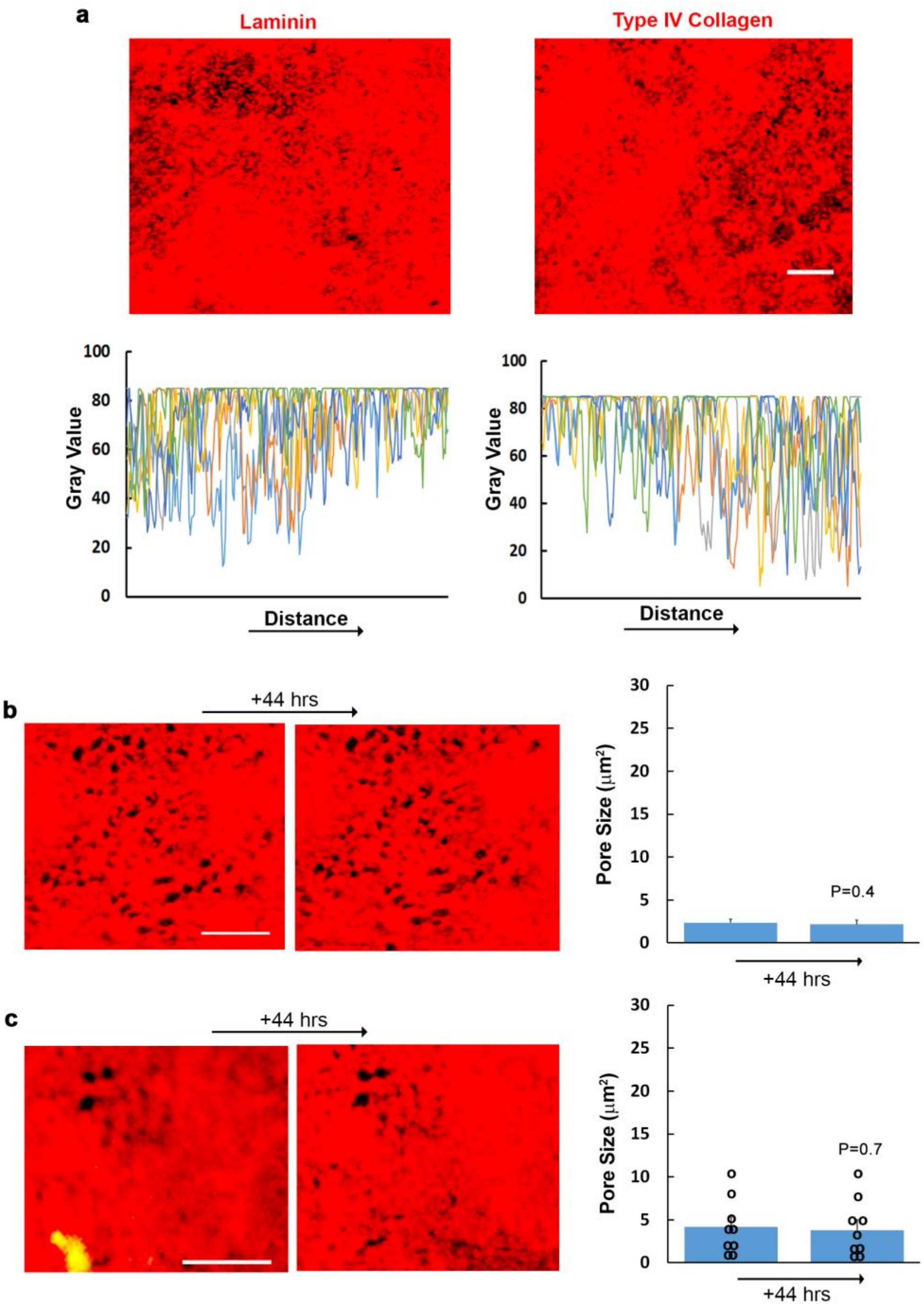

Supplementary Figure 3: Fluorescence intensity profiles of basement membrane pores. (A) Basement membranes of decellularized explants were immunostained for laminin (red) or type IV collagen (red) and normalized fluorescence intensity profiles for 6 randomly selected lines generated. (B) Pores in laminin-labeled explants cultured alone were imaged over a 44h time period and pore size quantified. Representative results of 3 experiment performed are shown and expressed as the mean ± SEM of 39 pores measured with significance determined by two-tailed Mann-Whitney U test. , ns; not significant. (C) Pores in laminin-labeled explants (red) at sites distant from labeled macrophages (yellow) were imaged over a 44h time period (at the end of the imaging period, macrophage fluorescence was bleached) and pore size quantified. Representative results from 3 experiments performed are shown and expressed as the mean ± SEM of 9 pores examined with significance determined by two-tailed Mann-Whitney U test. Bars: 10 µm.

Supplementary Figure 4

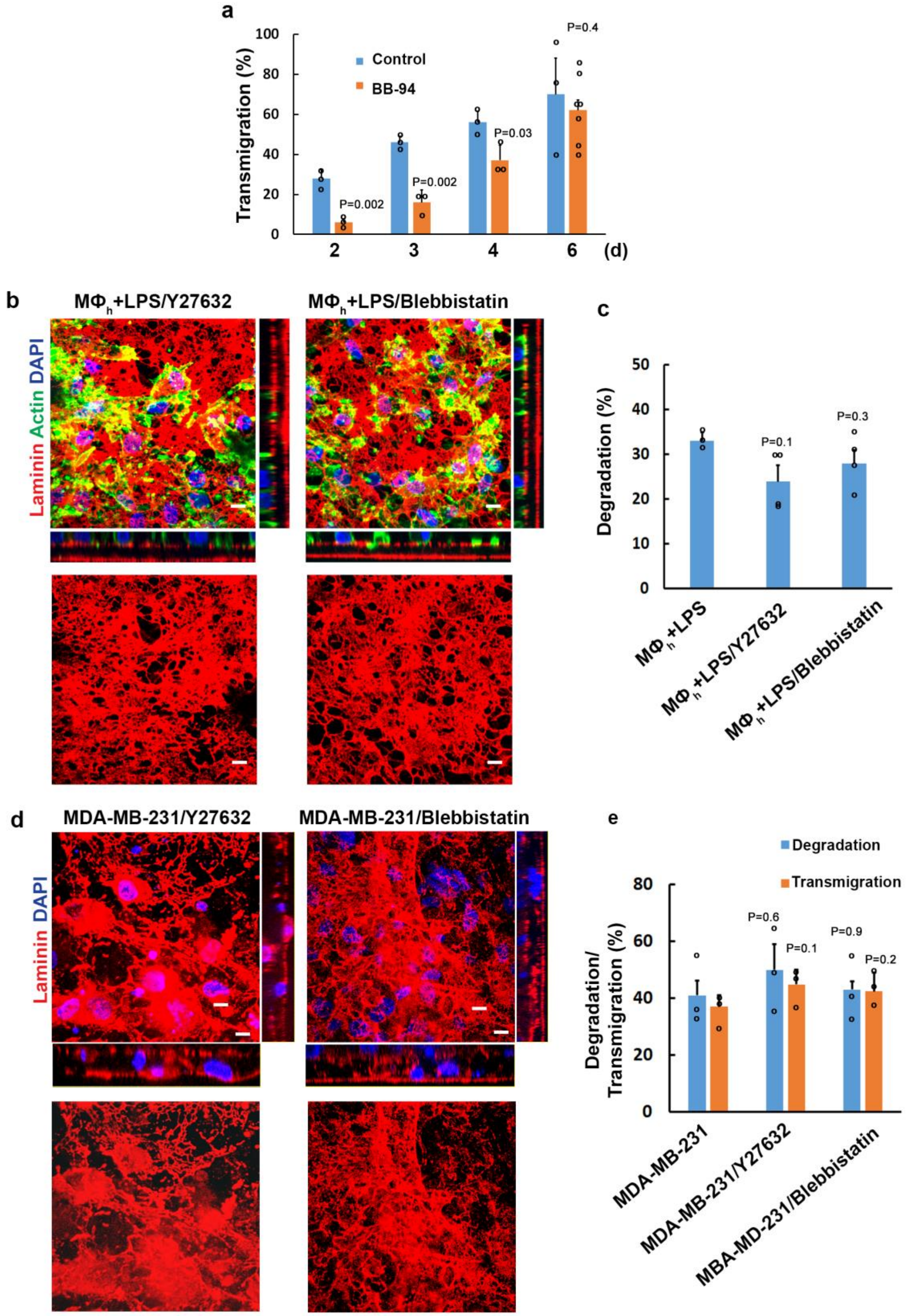

Supplementary Figure 4: Proteinase and actomyosin-dependent processes involved in macrophage and carcinoma cell transmigration/degradation. (A) The percent-transmigrated mouse macrophages was determined in the presence of the proteinase inhibitor cocktail with or without the addition of BB-94 as a function of time in culture. Results expressed as the mean ± SEM (n=3 independent exps) with significance determined by two-tailed t test. (B) En face and orthogonal immunofluorescence of basement membrane explants cultured with human macrophages polarized with LPS (1 µg/mL) in the absence or presence of Y-27632 (20 µM) (left panels) or blebbistatin (20 µM) (right panels). Images shown in lower are displayed without imaging associated macrophages to highlight basement membrane (red) degradation. Images shown are representative of 3 independent experiments. Bars: 10 µm. (C) The percent basement membrane degraded is quantified with results expressed as the mean ± SEM (n=3, 4, 4 independent exps for each of the listed variables) with significance determined by two-tailed t test. (D) En face and orthogonal immunofluorescence of basement membrane explants cultured with MDA-MB-231 carcinoma cells in the absence or presence of Y-27632 (20 µM) (left panels) or blebbistatin (20 µM) (right panels). Images shown in lower are displayed without imaging associated cancer cells to highlight basement membrane (red) degradation. Images shown are representative of 3 replicates. (E) The percent basement membrane degraded and the percent transmigrated MDA-MB-231 cells are quantified with results expressed as the mean ± SEM (n=3 independent exps) with significance determined by two-tailed t test.

Supplementary FIgure 5

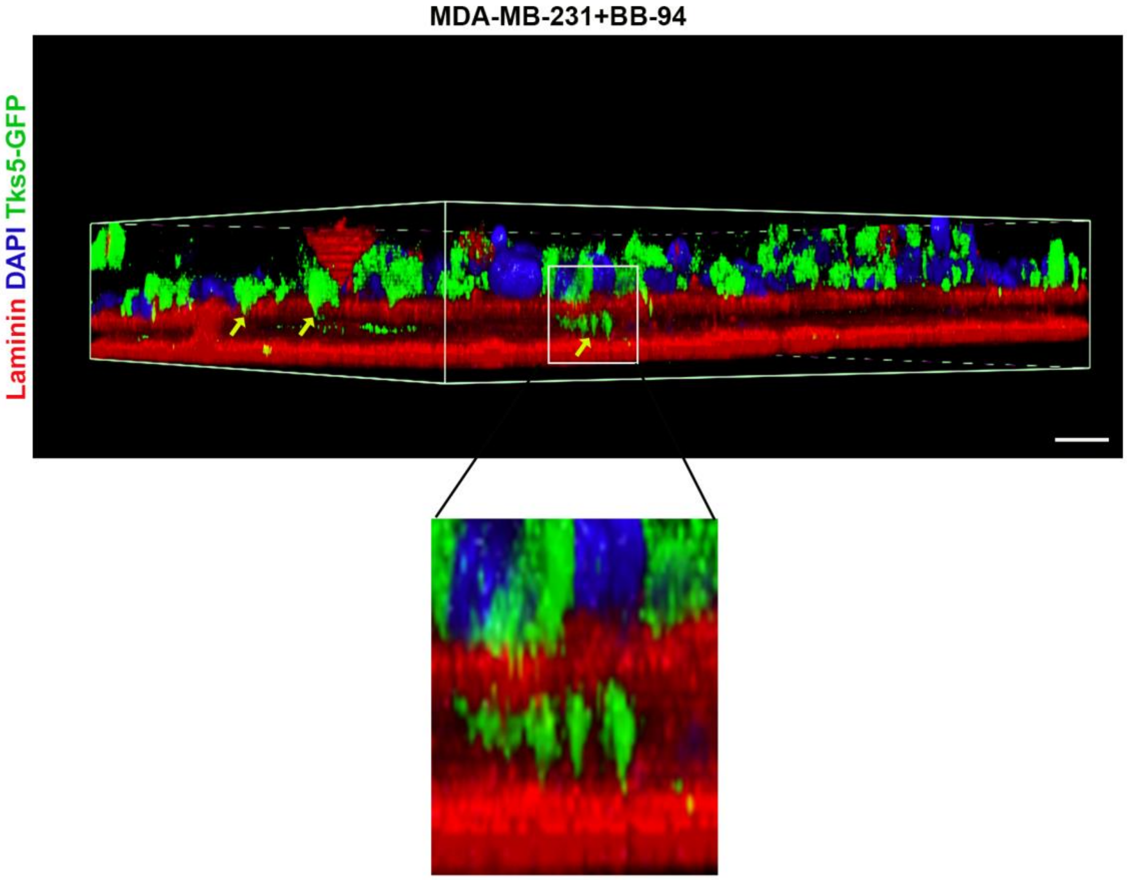

Supplementary Figure 5: MDA-MB-231 cells fail to transmigrate basement membrane in the absence of MMP activity. 3D reconstructions of Tks5-GFP transfected MDA-MB-231 (nuclei stained blue with DAPI) cultured in the presence of BB-94 atop laminin-labeled basement membranes (red) for 4 d extending cell protrusions through matrix pores while nuclei remain confined to the upper surface. Boxed area in upper image is expanded in the image shown below. Images are representative of 3 experiments performed. Bar: 10 µm.

Supplementary Figure 6

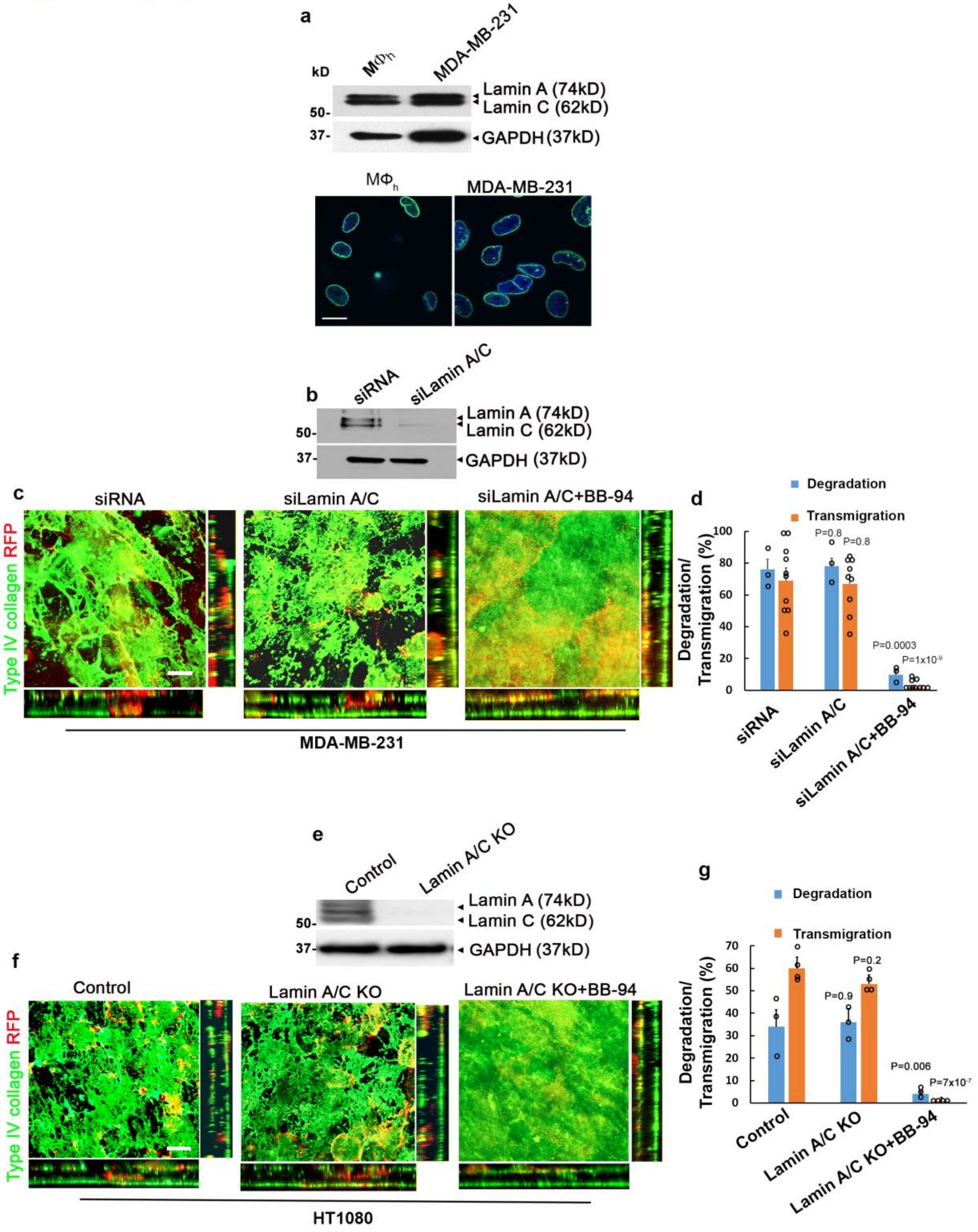

Supplementary Figure 6: Lamin A/C levels do not affect cancer cell basement membrane transmigration. (A) Equal numbers of human macrophages and MDA-MB-231 cells were either lysed and immunoblotted, or immunostained, to assess relative levels of lamin A/C (with nuclei counterstained blue with DAPI). Bar 10 µm. Results representative of 2 independent experiments performed. (B) Lamin A/C expression was silenced with a specific siRNA construct and the cells lysed prior to immunoblotting. Results representative of 3 independent experiments performed. (C) RFP-labeled MDA-MB- 231 cells were cultured atop basement membrane explants for 5 d and immunostained for type IV collagen (green) for en face or orthogonal imaging. Bar = 10 µm. (D) The percent basement membrane degraded and percent transmigrated cancer cells (control or lamin A/C siRNA-transfected MDA-MB-231 cancer cells) were quantified in the absence or presence of BB-94 (5 µM). Results are expressed as the mean ± SEM of 3 independent experiments for degradation and 9 experiments for invasion with significance determined by two-tailed t test. (E) Lamin A/C expression was targeted by CRISPR/Cas9 in HT-1080 cells and lysed prior to immunoblotting. Results representative of 2 experiments performed. (F) Control or lamin A/C knockout HT-1080 cells (red) were cultured atop basement membrane explants for 5 d and immunostained for actin (red) and type IV collagen (green) for en face or orthogonal imaging. Bar = 10 µm. Results representative of 3 independent experiments performed. (G) The percent basement membrane degraded and percent transmigrated HT-1080 cells were quantified in the absence or presence of BB-94 (5 µM). Results are expressed as the mean ± SEM (n=3 independent experiments for degradation and n=4 for transmigration) with significance determined by two-tailed t test.

Supplementary Figure 7:

Raw blot images from Supplementary Figure 6

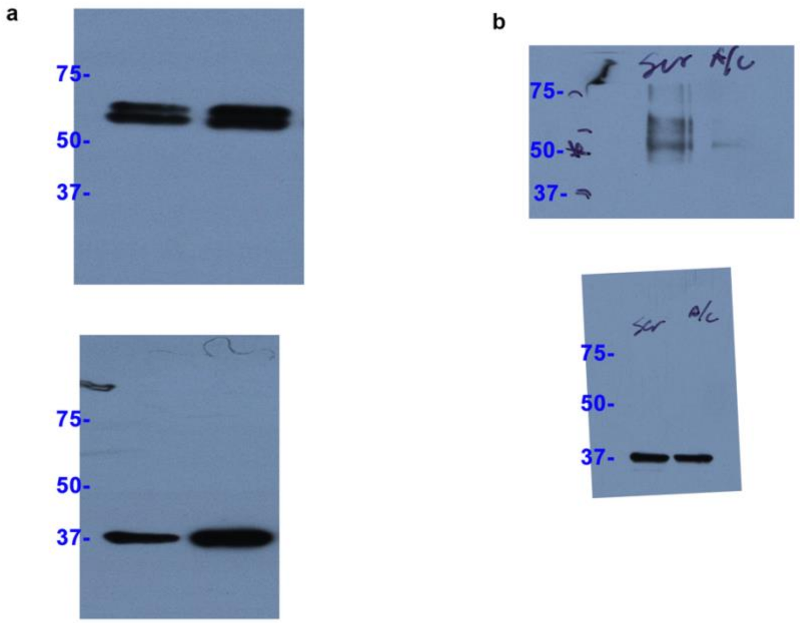

Supplementary Figure 7: Raw blot images corresponding to the supplementary panels indicated.

Supplementary Table 1: Antibodies used in this study

Antibody	Company	Catalog #	WB dilution	IF dilution	Blocking Dilution
Alpha 2(N) NC1 clone H22	Chondrex	#7071	1:1000	-	-
MT1-MMP	Abcam	ab53712	1:1000	1:500	-
Lamin A/C	CST	#2032	1:1000	1:500	-
GAPDH	CST	#4894	1:1000	-	-
b-actin	CST	#8457	1:1000	-	-
Laminin	Sigma	L9393	-	1:150	-
Type IV collagen	Abcam	ab19808	-	1:150	-
Elastin	EMD Millipore	#2039	-	1:150	-
TruStain FcX (anti-mouse CD16/32)	Biolegend	101309	-	-	10μg/ml
Human TruStain FcX	Biolegend	422301	-	-	1:20
Goat anti-Rabbit IgG, Alexa Fluor 488	Thermofisher	#A11008	-	1:1000	-
Goat anti-Rabbit IgG, Alexa Fluor 594	Thermofisher	#A32740	-	1:1000	-
Anti-rat IgG, HRP-linked Antibody	CST	#7077	1:2000	-	-
Anti-rabbit IgG, HRP-linked Antibody	CST	#7074	1:2000	-	-

Supplementary Table 2: qPCR primers used in this study

Target Gene	Forward Primer (5'-3')	Reverse Primer (5'-3')
MRC1 (human)	TGCTCTACAAGGGATCGGGT	ACACGCCAAACAAGAACATGA
TNFα (human)	ATGTTGTAGCAAACCCTCAAGC	TGATGGCAGAGAGGAGGTTG
MT1-MMP (human)	CAACATTGGAGGAGACACCCACT	CCAGGAAGATGTCATTTCCATTCA
GAPDH (human)	TCAAGGCTGAGAACGGGAAG	CGCCCCACTTGATTTTGGAG
Nos2 (mouse)	GAGACAGGGAAGTCTGAAGCAC	CCAGCAGTAGTTGCTCCTCTTC
Arg1 (mouse)	CATTGGCTTGCGAGACGTAGAC	GCTGAAGGTCTCTTCCATCACC
Gapdh (mouse)	ACTCCACTCACGGCAAATTC	TCTCCATGGTGGTGAAGACA
