# Supplementary material for: Divergent regulation of basement membrane trafficking by human macrophages and cancer cells
Source: Nat Commun. 2022 Oct 27;13:6409. doi: 10.1038/s41467-022-34087-x (PMC9613642; doi:10.1038/s41467-022-34087-x)
Supplement: Supplementary file 3 — Description of Additional Supplementary Files [file 41467_2022_34087_MOESM3_ESM.pdf]

Description of Additional Supplementary Files

Title: **Supplementary Data 1**
Description: Protease expression levels in MT1-MMP knockout versus control macrophages stimulated with LPS.

Title: **Supplementary Data 2**
Description: Red/Green single-color fluorescent images by figure.

Title: **Supplementary Movie 1**
Description: **3D rotation of rat mesentery basement membrane.** Laminin-stained (red) apical and reflected basal basement membrane surfaces in a 360° rotation. Depicted as a 3D-rendered confocal z-stack. Bar 20 µm.

Title: **Supplementary Movie 2**
Description: **3D rotation of rat mesentery interstitial matrix.** Elastin (blue) with second harmonic generation of type I collagen (yellow) in a 360° rotation. Depicted as a 3D-rendered confocal z-stack. Bar 20 µm.

Title: **Supplementary Movie 3**
Description: **Human macrophages dynamically protrude through the basement membrane.** 3D confocal time-lapse view of the interstitium-facing side of the apical basement membrane (red) as human macrophages (green) actively protrude through it. Time of observation: 160 min, captured every 10 min. Playback: 6 frames/s. Bar 20 µm.
