# Supplementary material for: Divergent regulation of basement membrane trafficking by human macrophages and cancer cells
Source: Nat Commun. 2022 Oct 27;13:6409. doi: 10.1038/s41467-022-34087-x (PMC9613642; doi:10.1038/s41467-022-34087-x)
Supplement: Supplementary file 5 — Supplementary Data 2 [file 41467_2022_34087_MOESM5_ESM.docx]

**Supplementary Data 2: Red/Green single-color fluorescence images by figure**
